# Phase Behavior of the Phytantriol/C_16_EO_10_ System under Controlled Hydration Conditions

**DOI:** 10.1021/acs.langmuir.6c02559

**Published:** 2026-07-20

**Authors:** Nicole Barber, Samina Akbar, Adam Squires

**Affiliations:** † Department of Chemistry, 1555 University of Bath, Bath BA2 7AY, U.K.; ‡ Department of Chemistry, University of Southampton, Southampton SO17 1BJ, U.K.

## Abstract

The phase behavior of the phytantriol/C_16_EO_10_/water system is investigated. This work shows that the addition of the surfactant C_16_EO_10_ (Brij C10) into the system allows the cubic diamond phase to exist at higher temperatures than for a system only containing phytantriol. The addition of C_16_EO_10_ also causes the water channels in the lipid structure to swell and as a result the cubic phases are shifted to be present at higher hydrations of up to 55% before reaching excess water. The temperature range was extended down to 0 °C, which further confirmed the gyroid and diamond phases are able to form below room temperature. This is a chemically robust system without any ester or C=C double bonds, unlike monoolein based systems. Our findings therefore have value for templating applications.

## Graphical abstract

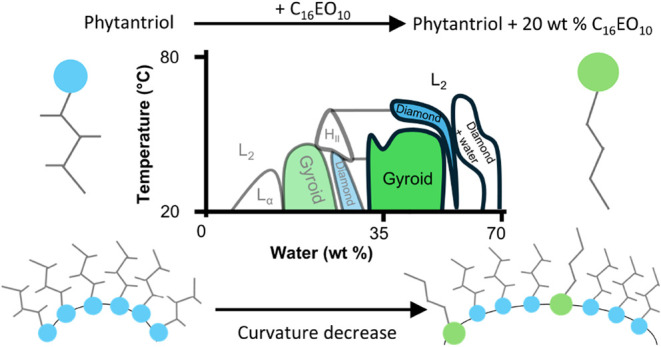

## Introduction

The cubic phases of lipids are nanomaterials^1^ that are formed by the self-assembly of amphiphilic molecules in the presence of water.^2^ They respond to changes in temperature and composition,^3^ which can lead to a wide range of applications. Examples of such applications include drug delivery,^4–6^ modeling of biological membranes,^7–9^ biosensors,^1,10^ and templating.^11–13^ There have been recent breakthroughs in using lipid cubic phases as anticancer drug delivery systems which have shown increased oral bioavailability and increased drug release rate.^14^ Encapsulating drugs in lipid cubic phases is known to protect them from degradation and increase the bioavailability.^15,16^ It can also increase the chemical stability against acid hydrolysis for sustained rather than triggered release in acidic environments such as the gastrointestinal tract.^17^ A greater understanding in controlling the size and structure of the lipid cubic phases could benefit the applications for drug release in future research where nanostructure impacts diffusion and release kinetics.^18^ Lipid cubic phases can also be used as tunable templates^12^ for metal deposition by introducing additives such as the surfactant C_16_EO_10_ (Brij C10). Systems using C_16_EO_10_ at 20 wt % in phytantriol have previously been studied at room temperature and in excess water conditions for templating applications.^12^ In the current manuscript, the effects of a fixed additive concentration of 20 wt % and temperature are investigated over a range of fixed hydration conditions using the phytantriol/C_16_EO_10_/water ternary system. This is a chemically robust system that at fixed hydration compositions allows access to a range of lattice parameter sizes as the nanostructure dimensions are transferred to the metal. This may be of interest in future research requiring a high level of control over nanostructure size.^19^

**Figure 1:**
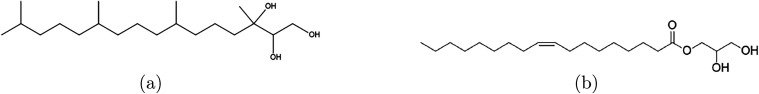
Structures of (a) phytantriol and (b) monoolein.

Phytantriol (3,7,11,15-tetramethylhexadecane-1,2,3-triol) (Figure 1a) is an amphiphilic molecule^20^ with a hydrophilic headgroup and a hydrophobic tail group; allowing it to self-assemble into diverse structures^21^ upon contact with water. The phase behavior of phytantriol under different hydration conditions has been previously reported by Barauskas et al.^3^ Phytantriol behaves in a similar way to monoolein (a commonly referenced lipid)^22–24^ which also forms inverse bicontinuous cubic phases as it acts as a type II lipid due to its bulkier tail group. Monoolein (Figure 1b) is a molecule that contains both an alkene and ester bond causing it to have high reactivity.^25^ Phytantriol has similar phase behavior to monoolein^3^ as both are amphiphilic molecules and form type II inverse structures due to their bulkier tail groups. However, phytantriol has only a hydrocarbon backbone with no C=C double bonds and no ester linkages, allowing it to maintain its self-assembled structures under harsher chemical conditions^20^ and is favored in electrodeposition processes to create thin films of metals.^26^ The addition of hexachloroplatinic acid to monoolein leads to a transition from cubic diamond to hexagonal phase, as shown in Figure S1 in the Supporting Information. This is likely due to acid-catalyzed hydrolysis of the ester linkage releasing un-ionized fatty acid^17^ which has previously been shown to favor a hexagonal phase.^27,28^

The phase behavior of phytantriol can be altered upon incorporation of additives such as surfactants.^12^ Addition of new additives can change the curvature of the structures formed by phytantriol and increase the temperature of the phase transitions.^29^ This has benefits of creating a broader range of structure sizes available for metal templating. Akbar et al.^12^ found that the incorporation of 20 wt % C_16_EO_10_ into the phytantriol/water system caused the lattice parameter of the lipid template to increase by approximately 5 nm^12^ compared to phytantriol only.

C_16_EO_10_ (Brij C10) (Figure 2) is a polyoxyethylene surfactant molecule. It has a long hydrocarbon tail and an ether link which make it chemically robust. Polyoxyethylenes have previously been used to template platinum on their own, resulting in the formation of hexagonal structures.^13,30^ It has a relatively small tail group compared to its headgroup and behaves as a type I surfactant. Its incorporation into a phytantriol/water system reduces the curvature of the lipid bilayer, resulting in the widening of the water channels.^12^

**Figure 2:**
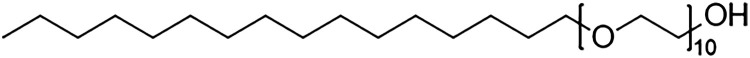
Structure of C_16_EO_10_.

There has been extensive research into the phase behavior of monoolein and adjacent monoglycerols.^22–24^ However, there is limited research into phase behavior of phytantriol;^3^ particularly with additives. Understanding changes in the structure of systems comprising of lipid, surfactant and water under different temperatures and hydration conditions can have benefits for applications such as templating, where it will allow greater control over the size of structures produced. Previous literature using the same ternary system as in this manuscript confirmed the cubic phase structures were able to form in both excess water and in excess hexachloroplatinic acid solution,^12^ indicating its suitability for templating. Furthermore, studies of binary systems of each of the two surfactant components, phytantriol^19^ and C_16_EO_10_ ^31^ at fixed hydration, show that each adopts the same structure with hexachloroplatinic acid as with water. Here we present the phase diagram and lattice parameter data for a phytantriol system with a fixed addition of 20 wt % surfactant (C_16_EO_10_) at various hydration levels and across a range of temperatures.

## Experimental Section

### Chemicals and Materials

Phytantriol (99.9% purity, UK) and C_16_EO_10_ were purchased from DSM Chemicals and Sigma-Aldrich, respectively. Deionized water purified using a Milli-Q system was used to create the fixed hydration pastes.

### Preparation of Fixed Hydration Pastes

A mixture of 80% phytantriol and 20% (w/w) C_16_EO_10_ was made by weighing appropriate amounts of phytantriol and C_16_EO_10_ into a glass vial. The vial was sealed and its contents heated to 80 °C for 15 min, followed by vortex mixing for approximately 1 min until achieving the appearance of a homogeneous, well mixed paste. A small amount of this mixture was added to a new glass vial and weighed, measured amounts of deionized water were then added to create fixed hydration pastes ranging from 5% up to 70% (w/w) hydration. Each vial was sealed with a lid and parafilm to ensure no evaporation of the water. The vials were then gently heated for 10 min and vortex mixed for 1 min or until a visible change in the lipid/water system was observed where the water and lipid had completely mixed and there was no longer a layer of water. The vials were then stored in the dark at room temperature for at least 2 weeks prior to analysis to allow the samples to equilibrate.

### Small Angle X-ray Measurements

SAXS measurements were performed using an AntonPaar SAXSpoint 2.0 instrument using a Cu–Kα X-ray source (1.54 Å). The samples were loaded in a multipaste holder for measurement. The temperature was varied between 0 to 80 °C, held for 30 min at each temperature for the sample to equilibrate. The diffraction patterns were recorded at a detector distance of 350 mm for 10 min. The data were reduced to 1D data using SAXS analysis software.

## Results and Discussion

The phases of the phytantriol/C_16_EO_10_/water ternary system were identified by small-angle X-ray scattering (SAXS) and are presented as a phase diagram in Figure 3. Representative SAXS patterns of each phase with labeled peaks are presented in the Supporting Information (Figures S2–S4). Full SAXS patterns for each hydration paste are also presented in Supporting Information. At low hydration levels (below 25%) the ternary system exists in an inverse micellar phase (*L*_2_). This means there is a lack of ordered, long-range structure in the system. In mid range hydration levels (25–50%) the system organizes into a cubic gyroid phase (*Q* ^230^) and transitions through a cubic diamond (*Q* ^224^) back into micellar at higher temperatures. The highest hydration levels from 55 to 70% show the cubic diamond phase dominates. The 55% hydration level shows the diamond phase exists without excess water at temperatures of 25 °C and below. Regions of excess water can be identified from the lattice parameter data shown in Figure 4b, where the lattice parameter no longer increases with increasing water content. Low temperatures cause the water channels to widen due to the relaxation of the lipid molecules.^29^ This allows for more water to be taken into the structure before reaching excess hydration. Quantitative lattice parameter data is shown in Figure 4, indicating the changes in lattice parameter with temperature. At the hydration content of 55% above 25 °C the water channels constrict to a size at which no more water can be taken into the structure; leading to the cubic diamond phase existing in excess water. The diamond phase for the phytantriol/C_16_EO_10_/water system exists at higher hydration levels than a system of only phytantriol and water which was found to exist at 26–28% hydration.^3^ This allows a higher fill fraction of aqueous media in the lipid template.

**Figure 3:**
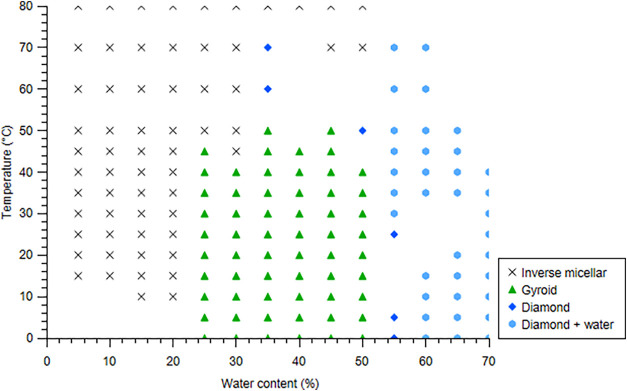
Phase diagram of the phytantriol/C_16_EO_10_/water system.

**Figure 4:**
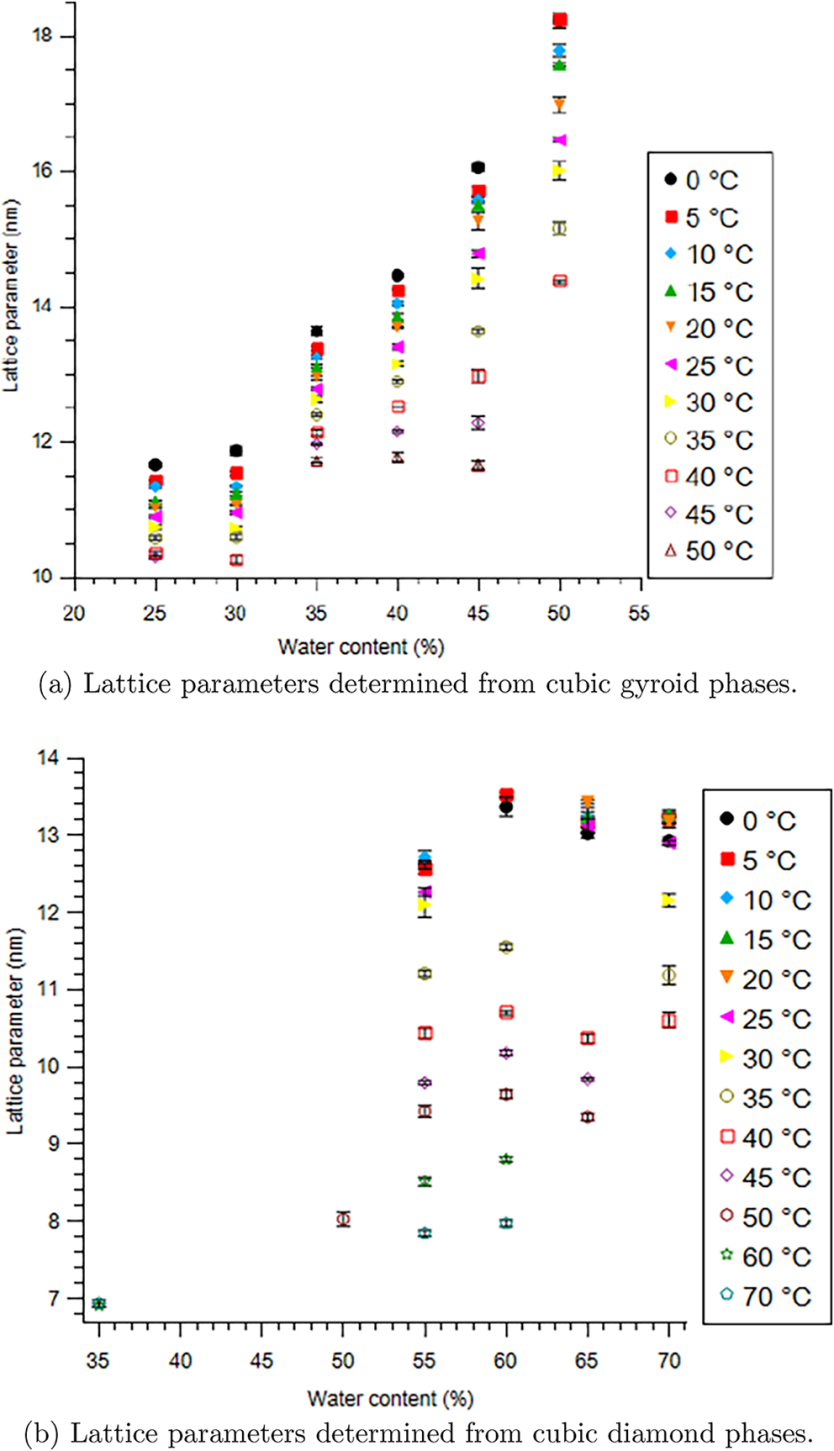
Lattice parameters determined for (a) gyroid phases and (b) diamond phases.

The data presented in this phase diagram confirm the existence of bicontinuous cubic phases with the addition of 20 wt % C_16_EO_10_, as expected.^12^ In addition to expanding the water content range of the cubic phases, the temperature range is also expanded. This allows the cubic diamond phase to exist at temperatures up to 70 °C. In this study we increased the temperature range to extend below room temperature; this has resulted in finding that both cubic phases of diamond and gyroid can exist at low temperatures. Previous research using monoacylglycerols as the lipid systems in water have shown that the gyroid phase is stable down to temperatures of −10 °C,^32^ while other research has confirmed the presence of the diamond cubic phase of phytantriol in controlled, low hydration conditions down to temperatures of −120 °C.^33^ The importance of maintaining these cubic structures at low temperatures comes from research in drug delivery and protein crystallization. By maintaining a cubic phase at a low temperature this provides a way of storing drugs before carrying out drug release studies. A further use is allowing researchers to crystallize and study protein membranes which might otherwise degrade at room temperature.^32^

The changes in the calculated lattice parameters for both the gyroid phases and the diamond phases in the phytantriol/C_16_EO_10_/water system are presented in Figure 4.

From Figure 4a it is evident that increasing the hydration level leads to increasing lattice parameter, as a higher water content means more water for the lipid system to take in. Temperature has the opposite effect on lattice parameter as increasing the temperature causes the lattice parameter to decrease. This is due to an increase in lipid chain disorder causing the tail groups splay out more at higher temperatures and the water channels to constrict.^34^ Lattice parameter sizes are achieved ranging from 10 to 18 nm by controlling the water content and the temperature. This is a much greater range than is seen for the binary phytantriol system in which the gyroid phase exhibits lattice parameters between 8.6 and 10 nm.^3^ This indicates that a fine control over both these factors creates a wider range of sizes available for templating applications. Controlling the lipid template size for creating metal nanostructures could improve stability of the resulting metal catalysts by tuning the thickness of the nanowires formed. This can also influence the selectivity toward some reactions such as the oxygen reduction reaction in fuel cells.^12,35^

Figure 4b presents the changes in the calculated lattice parameters of the cubic diamond phases. The graph presents a similar trend to that of the gyroid phases with water content increasing the lattice parameter and temperature decreasing the lattice parameter. The lattice parameter for the phytantriol only system has previously been reported as 7.0 ± 0.2 nm^12^ compared to the values of 12–13 nm that are seen in this study for the system with additional C_16_EO_10_ in excess water conditions at room temperature. These values are in good agreement with values previously measured for this system under excess water conditions and at room temperature.^36^ This increased range in lattice parameter of the cubic diamond phase could have applications in drug delivery methods as a tunable pore size may allow different rates of drug release.^37^ This could also be utilized to store larger molecules such as proteins by using the additive to swell the lipid structure to a larger size.^37^ The points at which the lattice parameter no longer increases with water content and are only controlled by the changes in temperature confirm the system has reached excess hydration. This can be seen to occur from 60% water content at low temperatures and from 55% at higher temperatures.

## Conclusion

In summary, we have presented the phase diagram and changes in lattice parameters for the phytantriol/C_16_EO_10_/water system. This confirms the phase behavior is similar to that of the phytantriol/water system but the addition of C_16_EO_10_ increases the stability of the bicontinuous phases at higher temperatures and also causes the water channels in the phytantriol structure to swell. This allows a greater intake of water into the lipid system and leads to the cubic phases being present at higher hydration levels without reaching excess water. These features have benefits for templating applications: the system is chemically robust, and the higher temperature range allows access to a broader range of sizes than phytantriol alone. The addition of C_16_EO_10_ retains the chemical stability of the lipid/water system, and so the larger water channel sizes may allow for applications involving larger molecules.

## Supplementary Material



## Notes

Materials and methods for monoolein in excess HCPA and water; indexed SAXS patterns of gyroid and diamond phases; SAXS patterns for all samples measured; lattice parameter data for gyroid and diamond phases (PDF)

## Abbreviations Used

SAXS: small angle X-ray scattering
LCP: lipid cubic phase
