# Supplementary material for: Phase Behavior of the Phytantriol/C16EO10 System under Controlled Hydration Conditions
Source: Langmuir. 2026 Jul 20;42(30):22209–13. doi: 10.1021/acs.langmuir.6c02559 (PMC13450430; doi:10.1021/acs.langmuir.6c02559)
Supplement: Supplementary file 1 [file la6c02559_si_001.pdf]

Phase behaviour of the phytantriol/$C_{16}EO_{10}$ system under controlled hydration conditions

Nicole Barber,[†] Samina Akbar,[‡] and Adam Squires[*,†]

†*Department of Chemistry, University of Bath, Bath, BA2 7AY*

‡*Department of Chemistry, University of Southampton, Southampton, SO17 1BJ*

E-mail: a.squires@bath.ac.uk

Contents

Experimental method and data on monoolein in HCPA **S2**

Representative SAXS patterns of micellar, gyroid and diamond phases **S3–S5**

SAXS patterns of hydration pastes over temperature range **S6–S12**

Lattice parameter data of gyroid and diamond phases **S13**

Experimental

Chemicals and materials

Hexachloroplatinic acid (HCPA, 8 wt% in water) was used as purchased from Sigma-Aldrich. Monoolein (cithrol) was borrowed from the University of Reading.

Methods

A hydration paste was made by weighing an appropriate amount of monoolein into a glass vial. An appropriate amount of DI water and HCPA was added to each of the vials of monoolein to create an excess of hydration as determined from the phase diagrams presented Kulkarni et al. 2011. The pastes were heated gently to 50 °C and vortex mixed for 1 minute until a homogenous paste was achieved. The pastes were then left for 24 hours before running analysis.

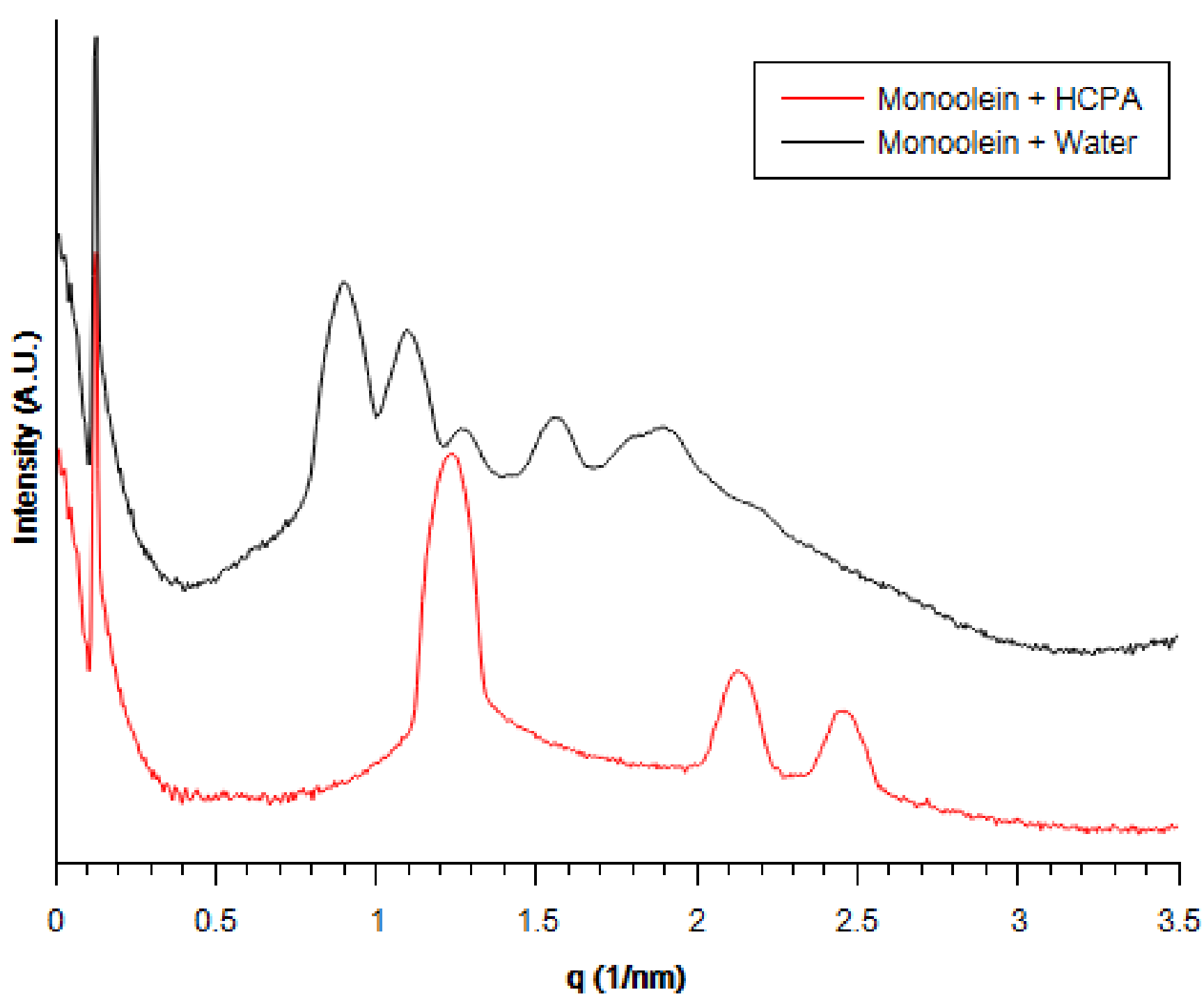

Figure S1: SAXS patterns of monoolein in excess HCPA and water at 25 °C.

Below are representative SAXS patterns of the phases described in the phase diagram.

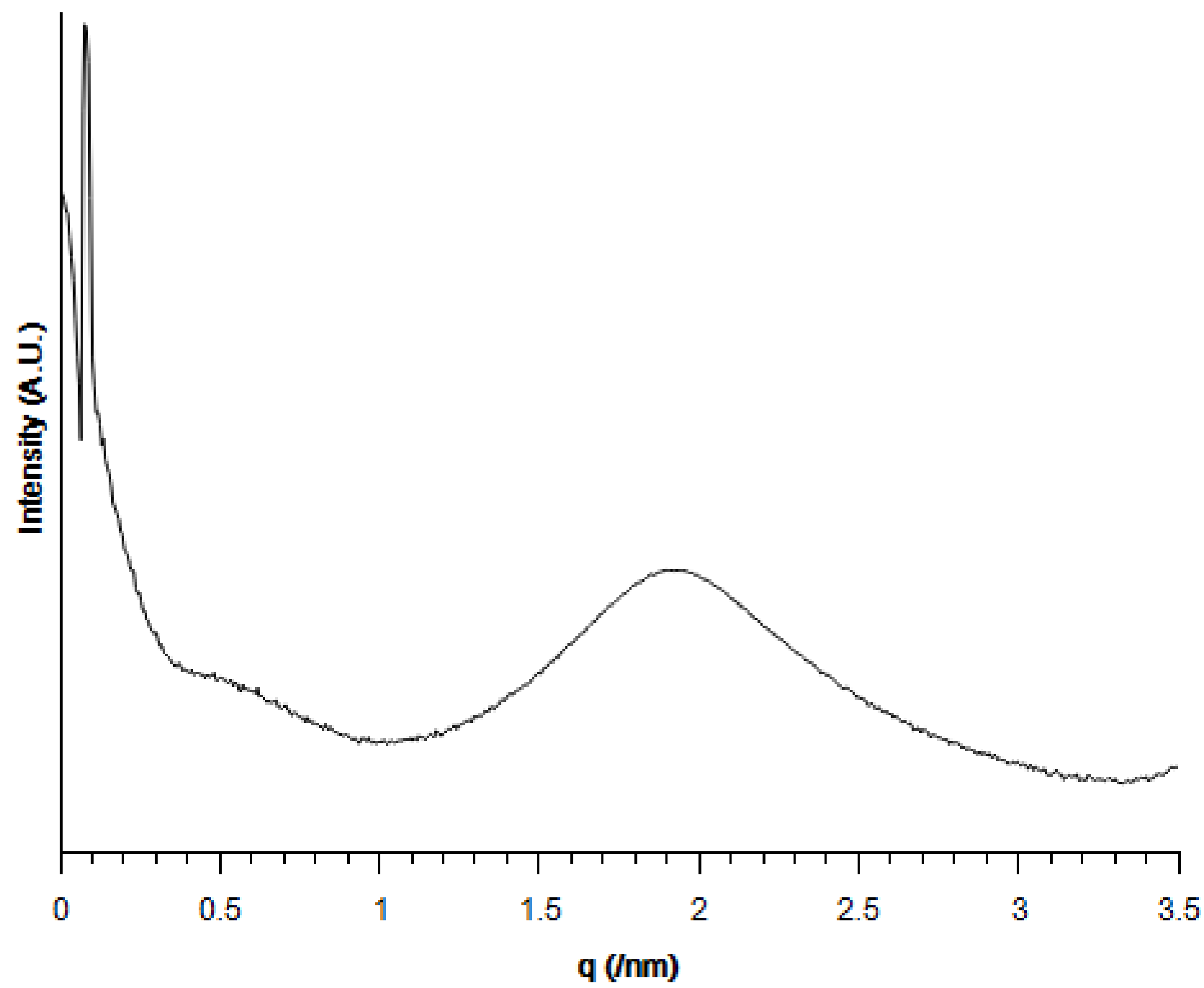

Figure S2: Representative inverse micellar SAXS pattern from sample at 15% water content and 25 °C.

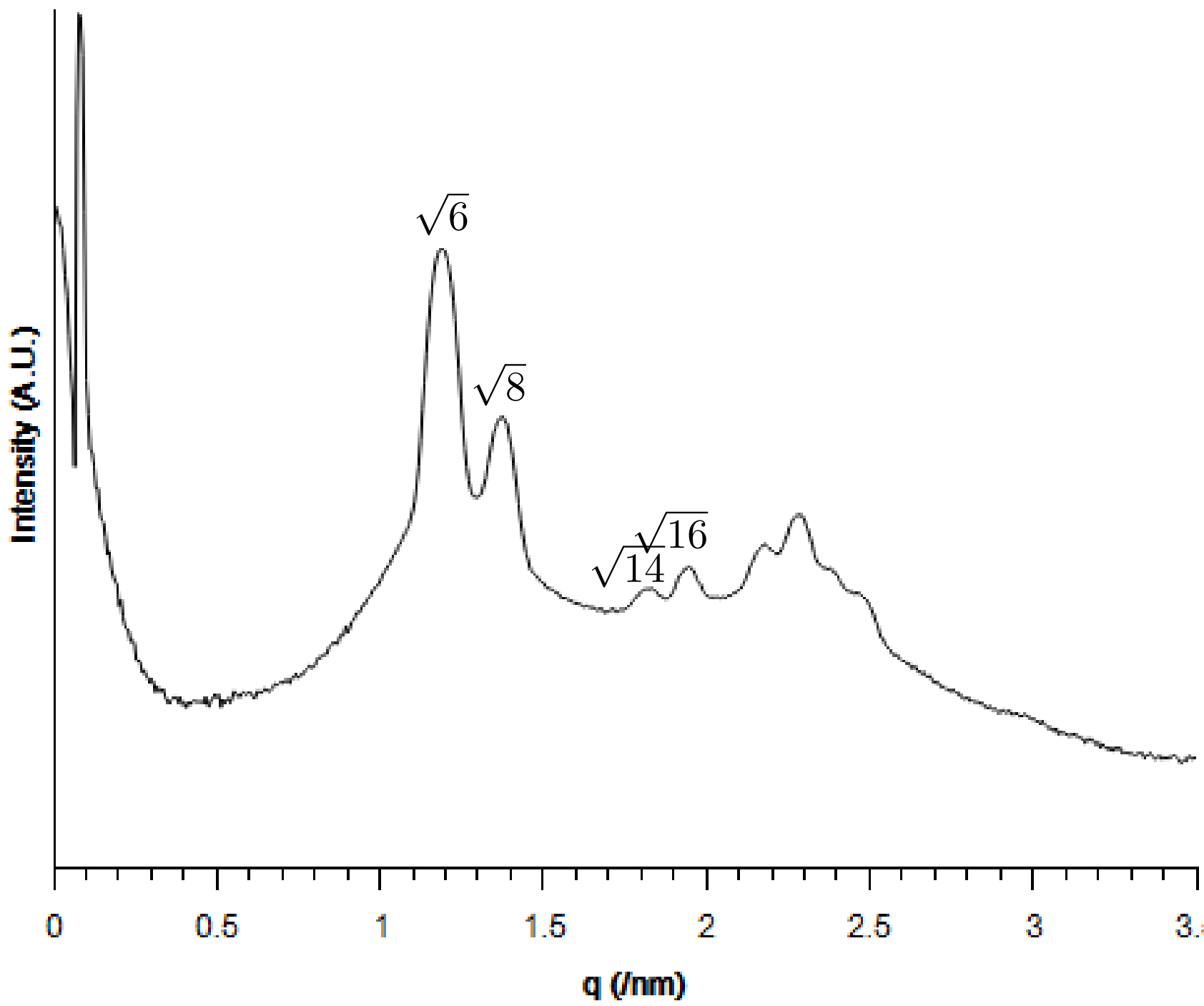

Figure S3: Representative cubic gyroid SAXS pattern from sample at 35% water content and 25 °C.

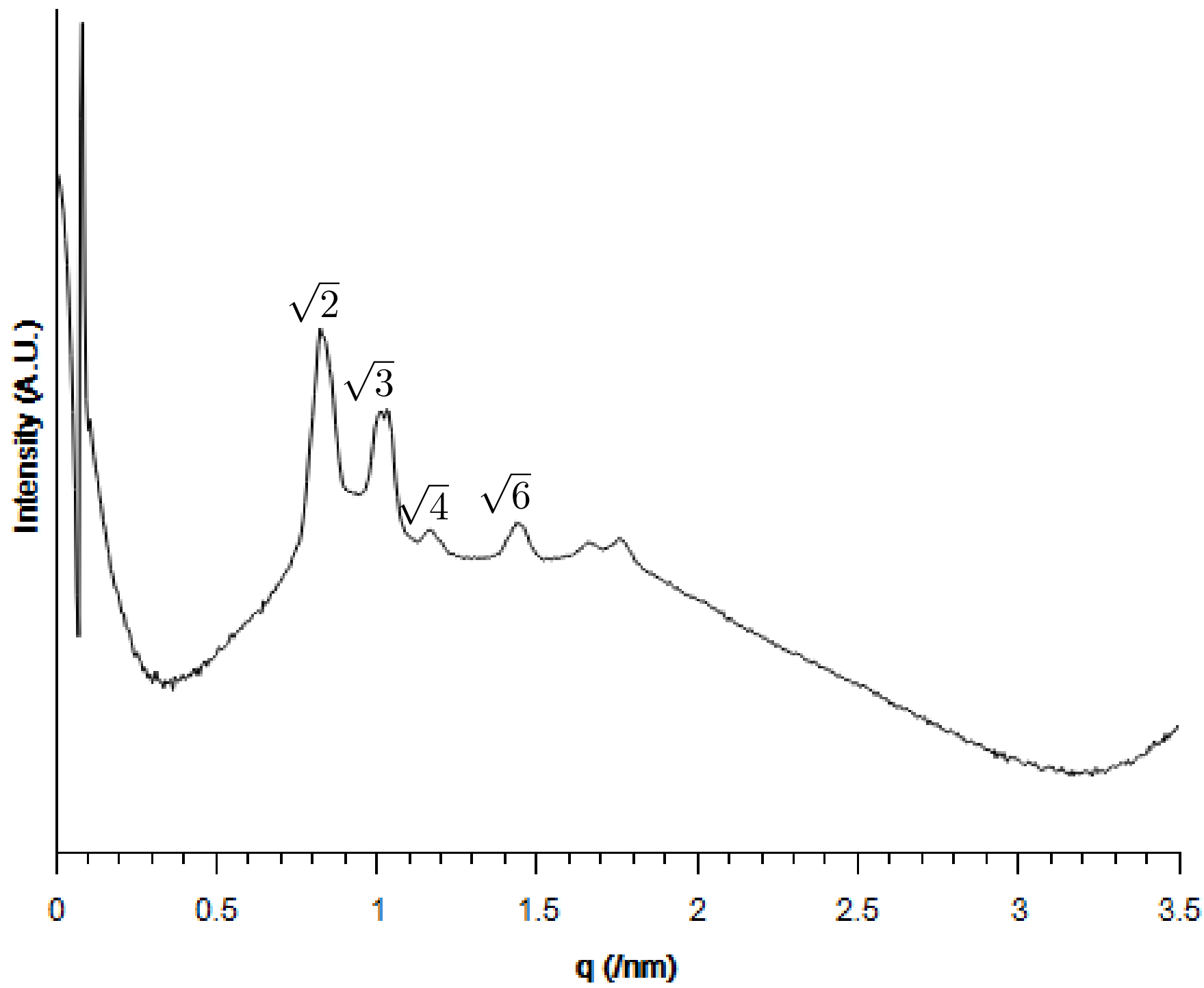

Figure S4: Representative cubic diamond SAXS pattern from sample at 55% water content and 45 °C.

The following are SAXS patterns collected for each hydration paste across the temperature range.

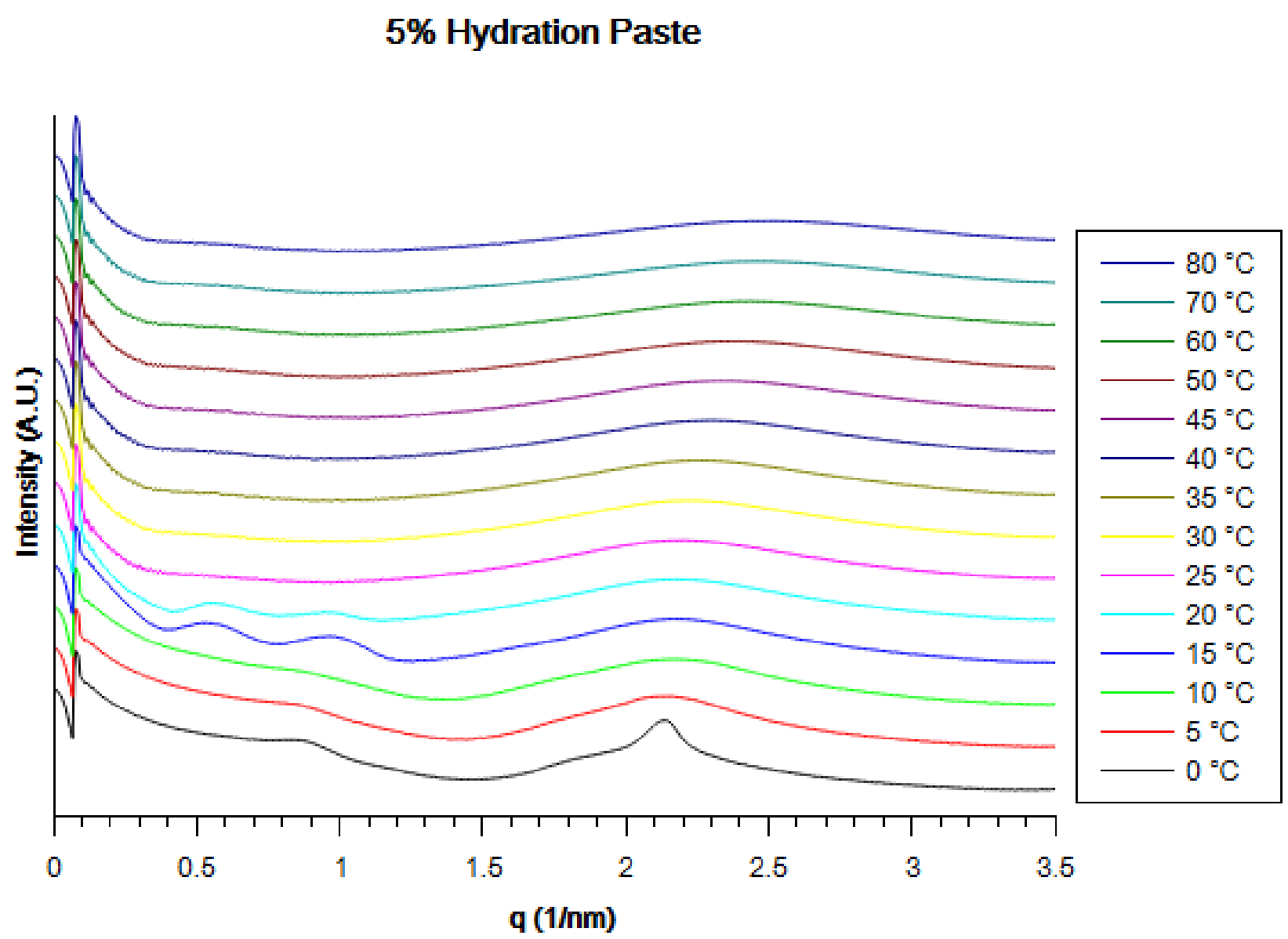

Figure S5: SAXS patterns from 5% hydration paste over the temperature range.

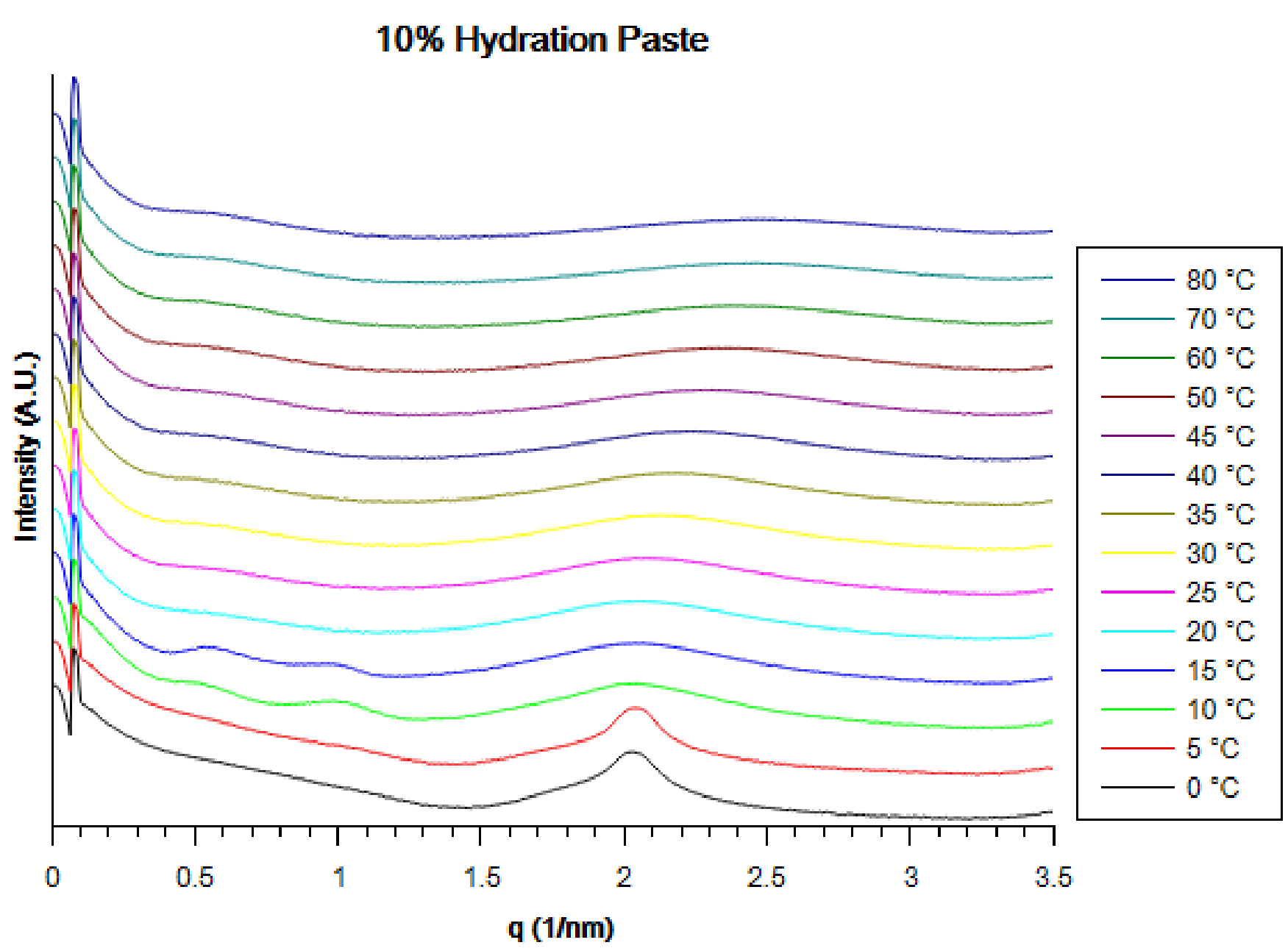

Figure S6: SAXS patterns from 10% hydration paste over the temperature range.

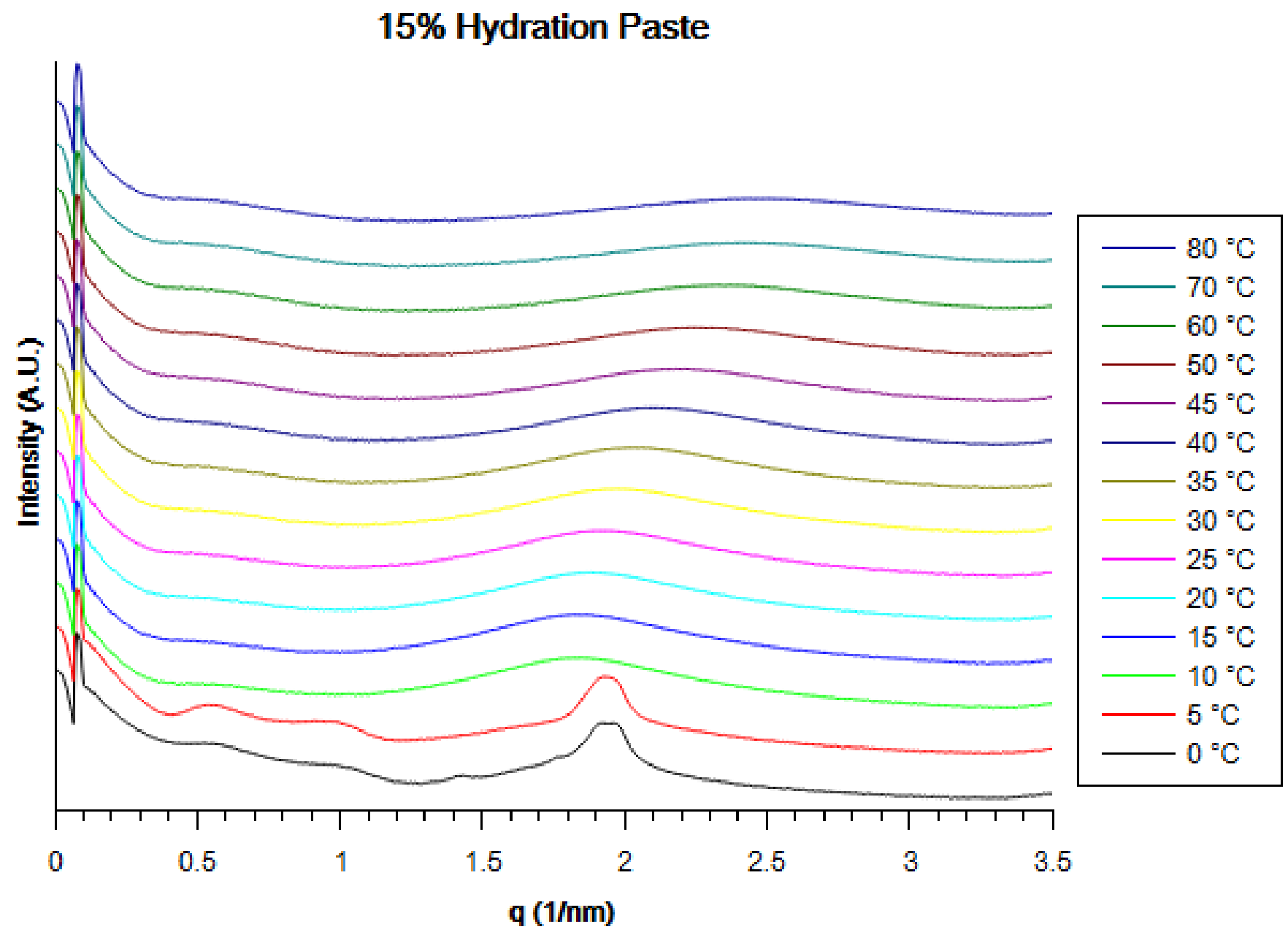

Figure S7: SAXS patterns from 15% hydration paste over the temperature range.

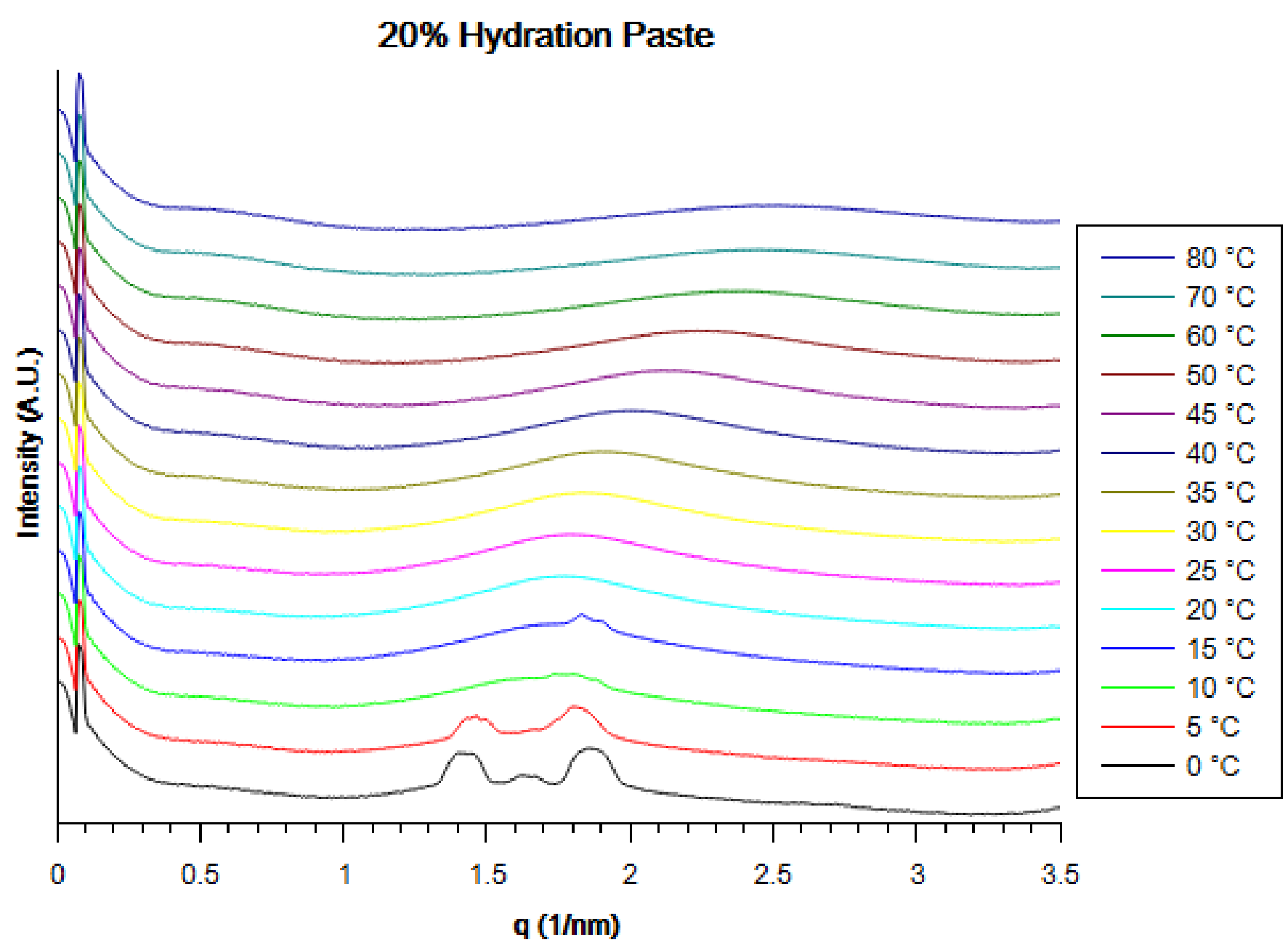

Figure S8: SAXS patterns from 20% hydration paste over the temperature range.

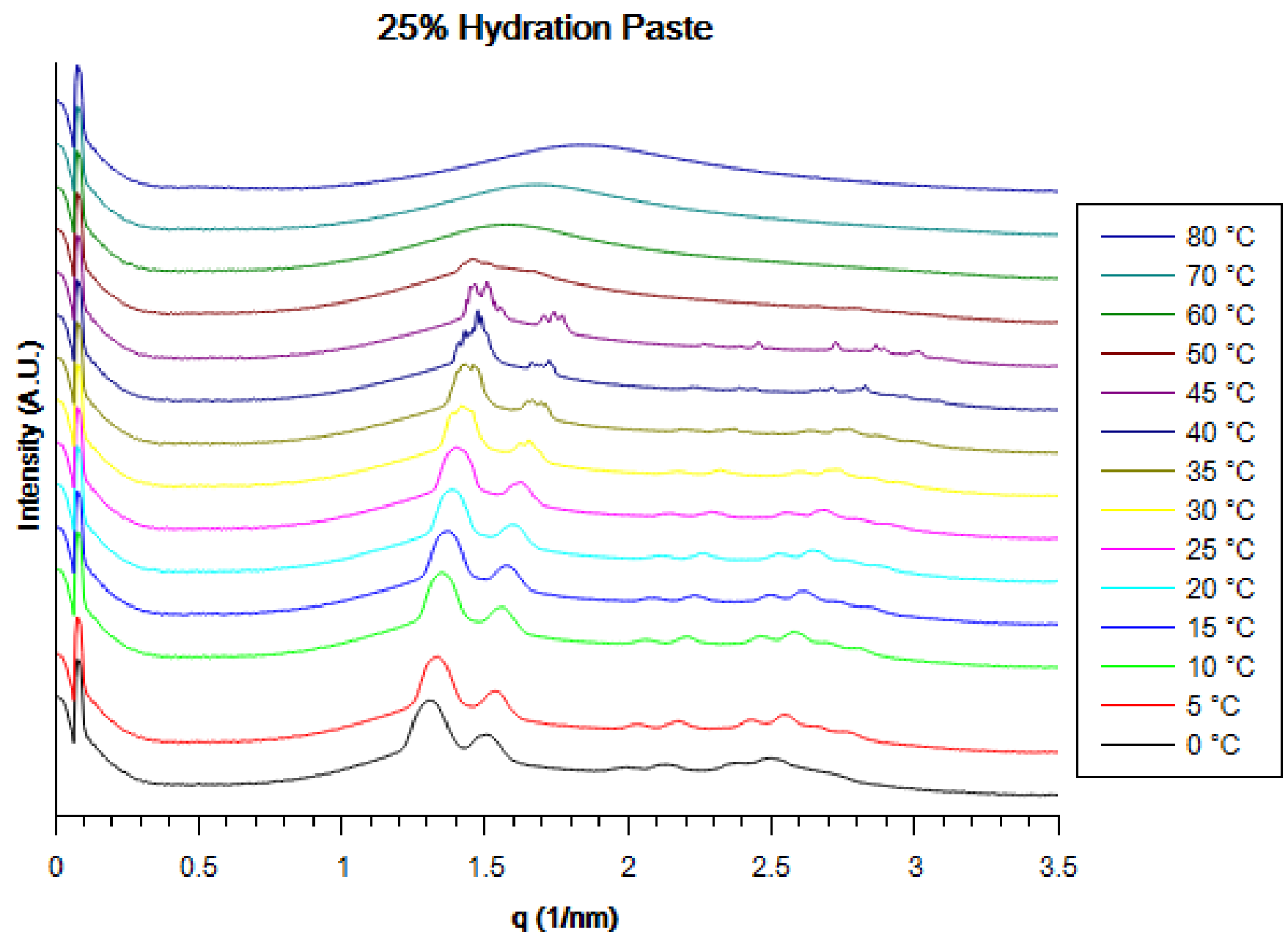

Figure S9: SAXS patterns from 25% hydration paste over the temperature range.

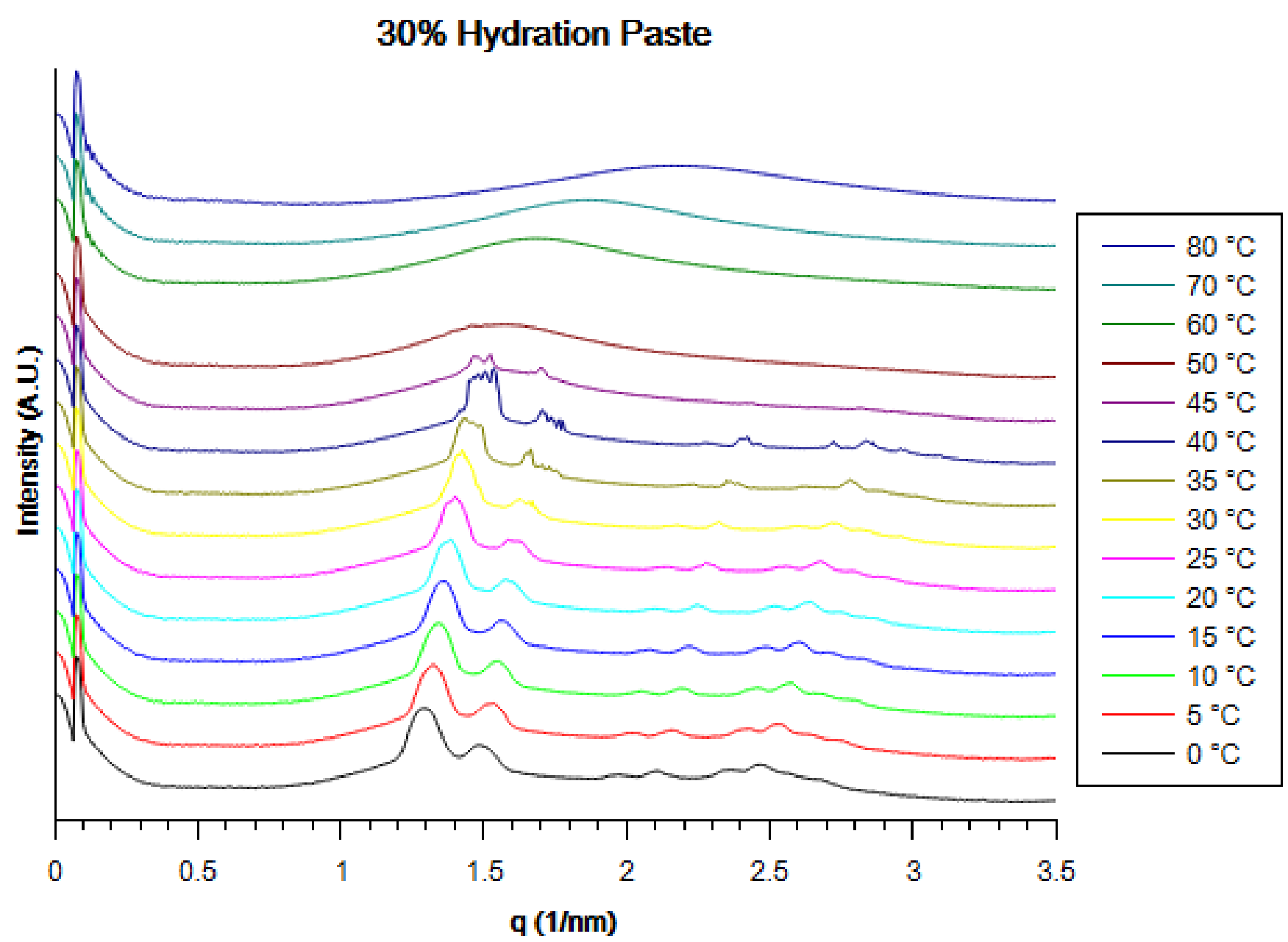

Figure S10: SAXS patterns from 30% hydration paste over the temperature range.

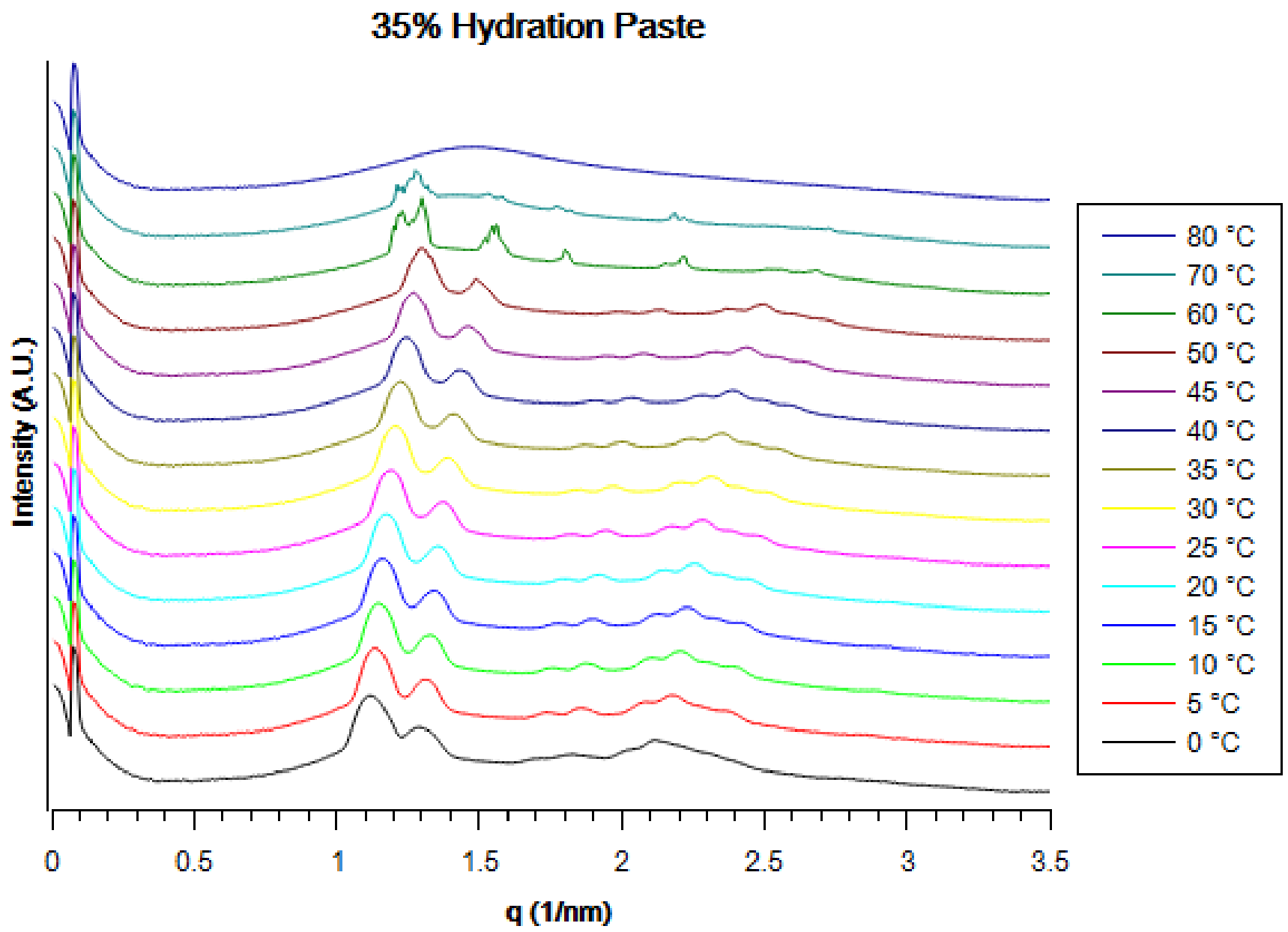

Figure S11: SAXS patterns from 55% hydration paste over the temperature range.

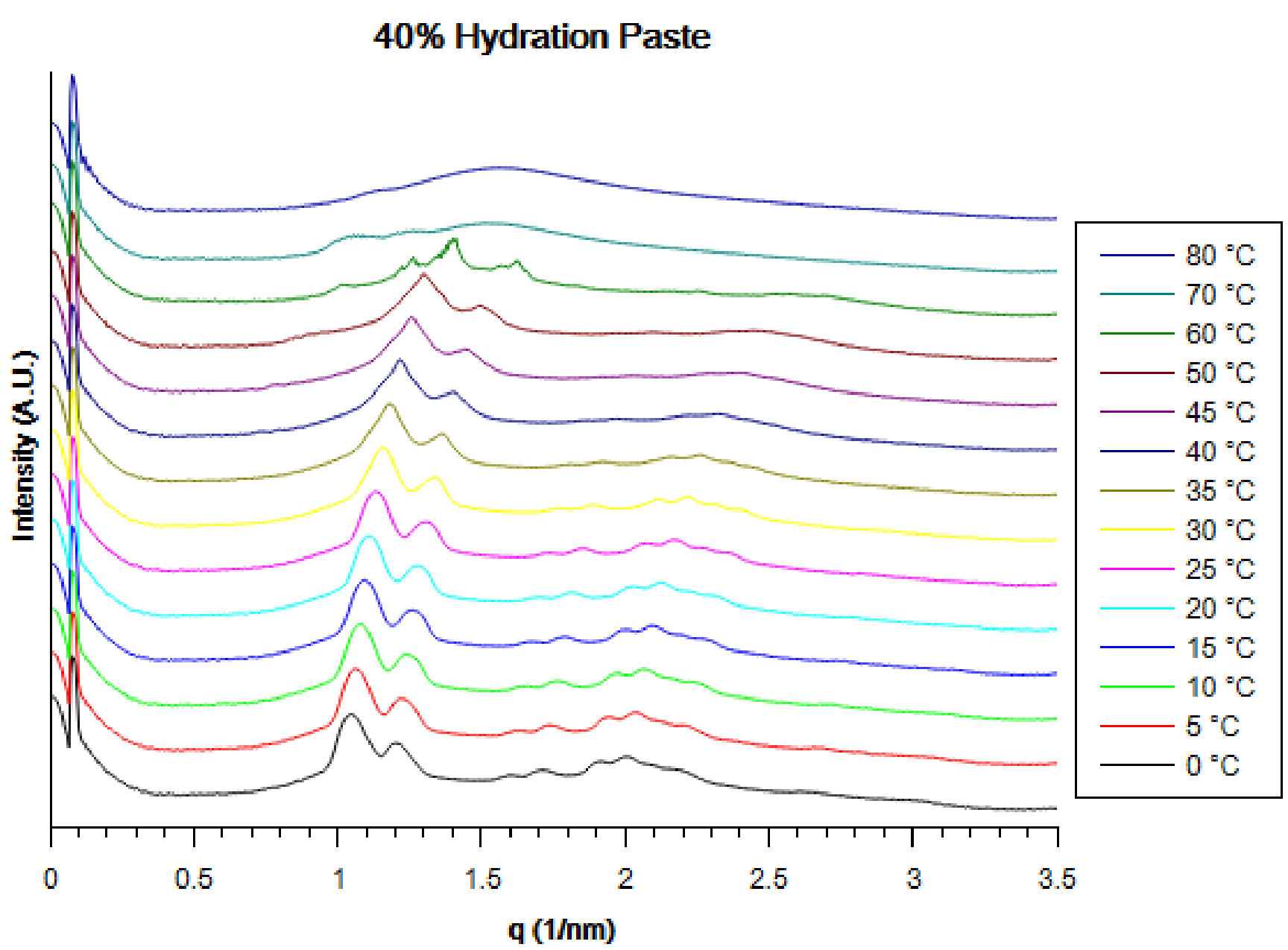

Figure S12: SAXS patterns from 40% hydration paste over the temperature range.

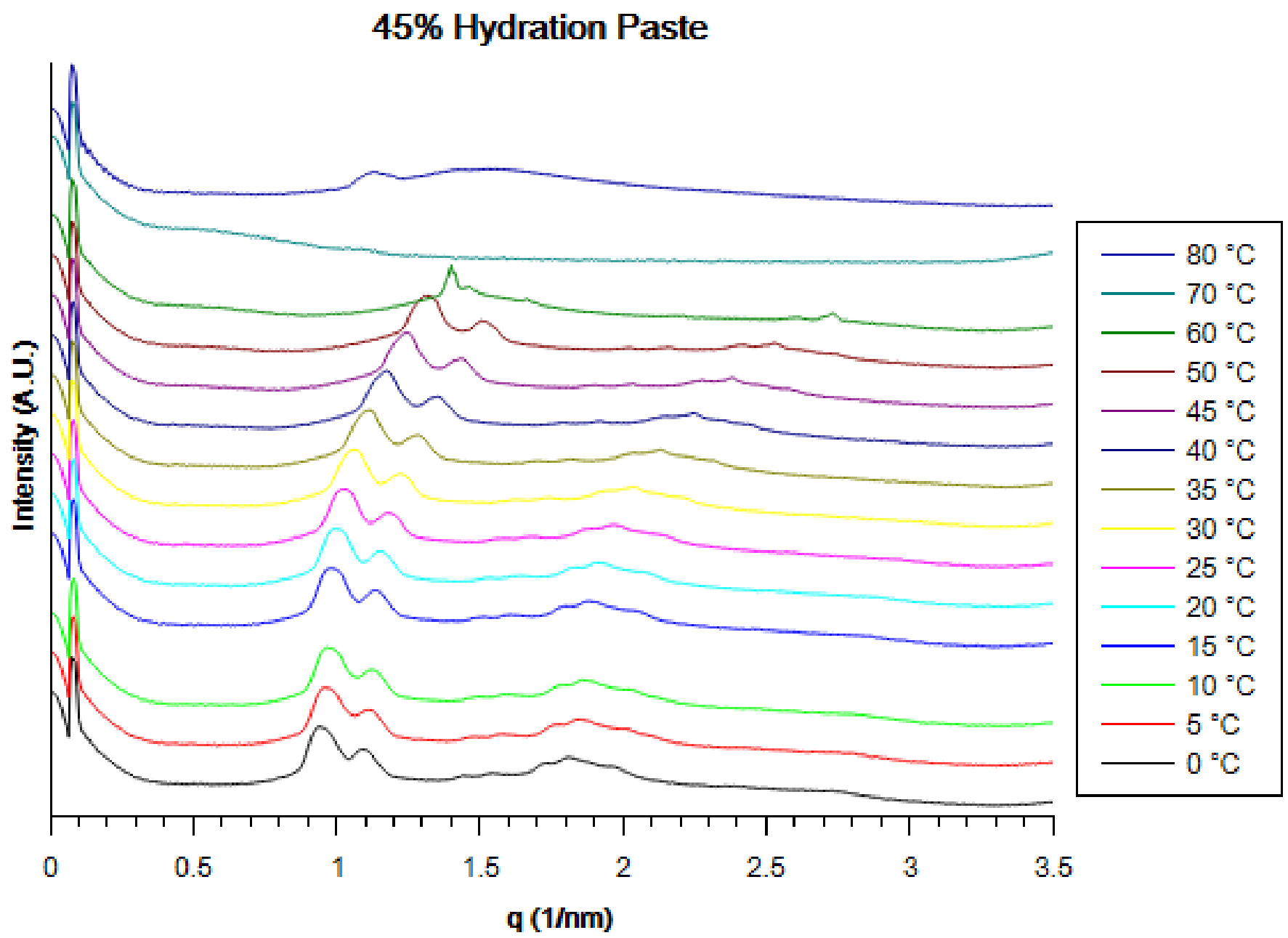

Figure S13: SAXS patterns from 45% hydration paste over the temperature range.

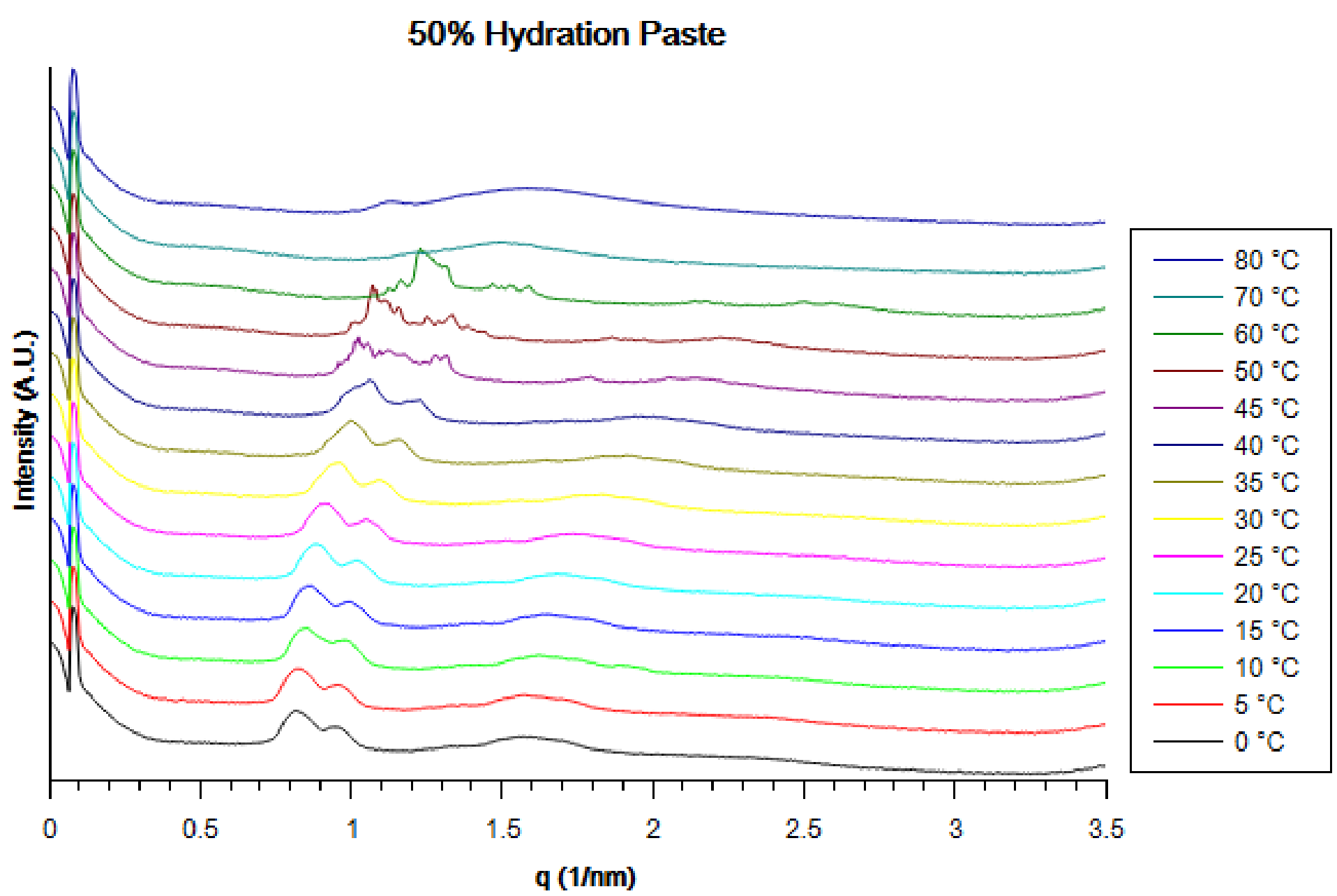

Figure S14: SAXS patterns from 50% hydration paste over the temperature range.

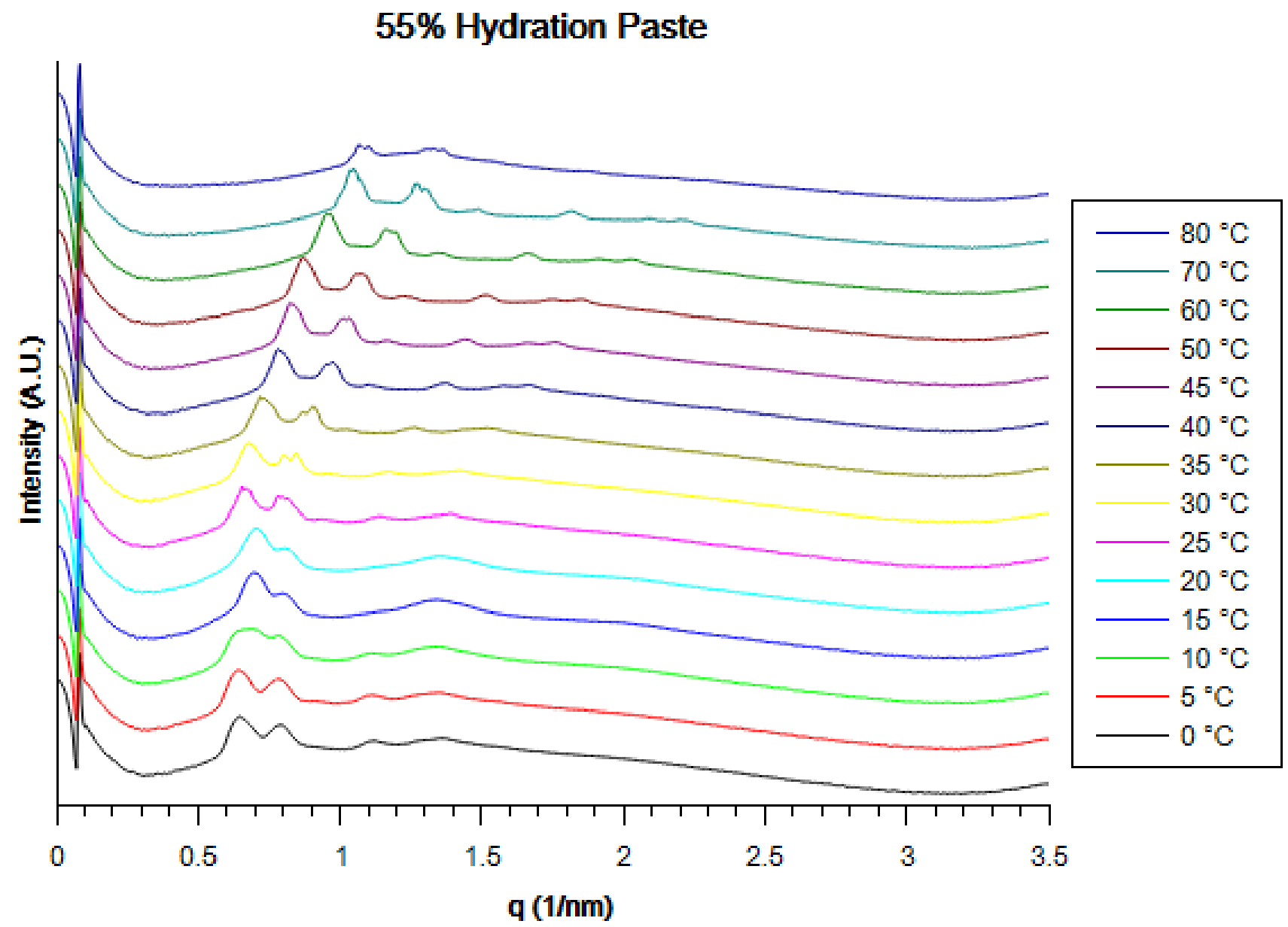

Figure S15: SAXS patterns from 55% hydration paste over the temperature range.

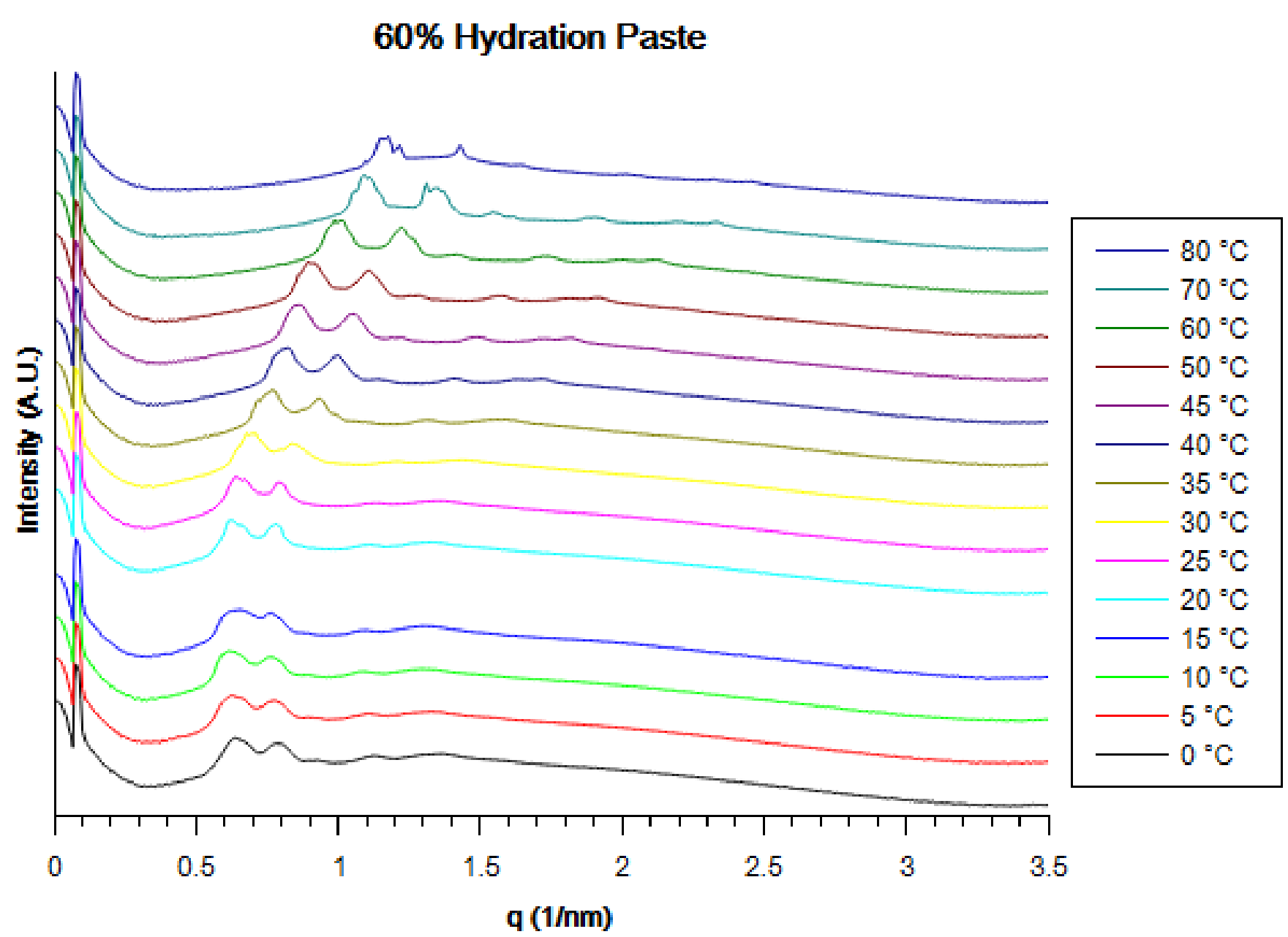

Figure S16: SAXS patterns from 60% hydration paste over the temperature range.

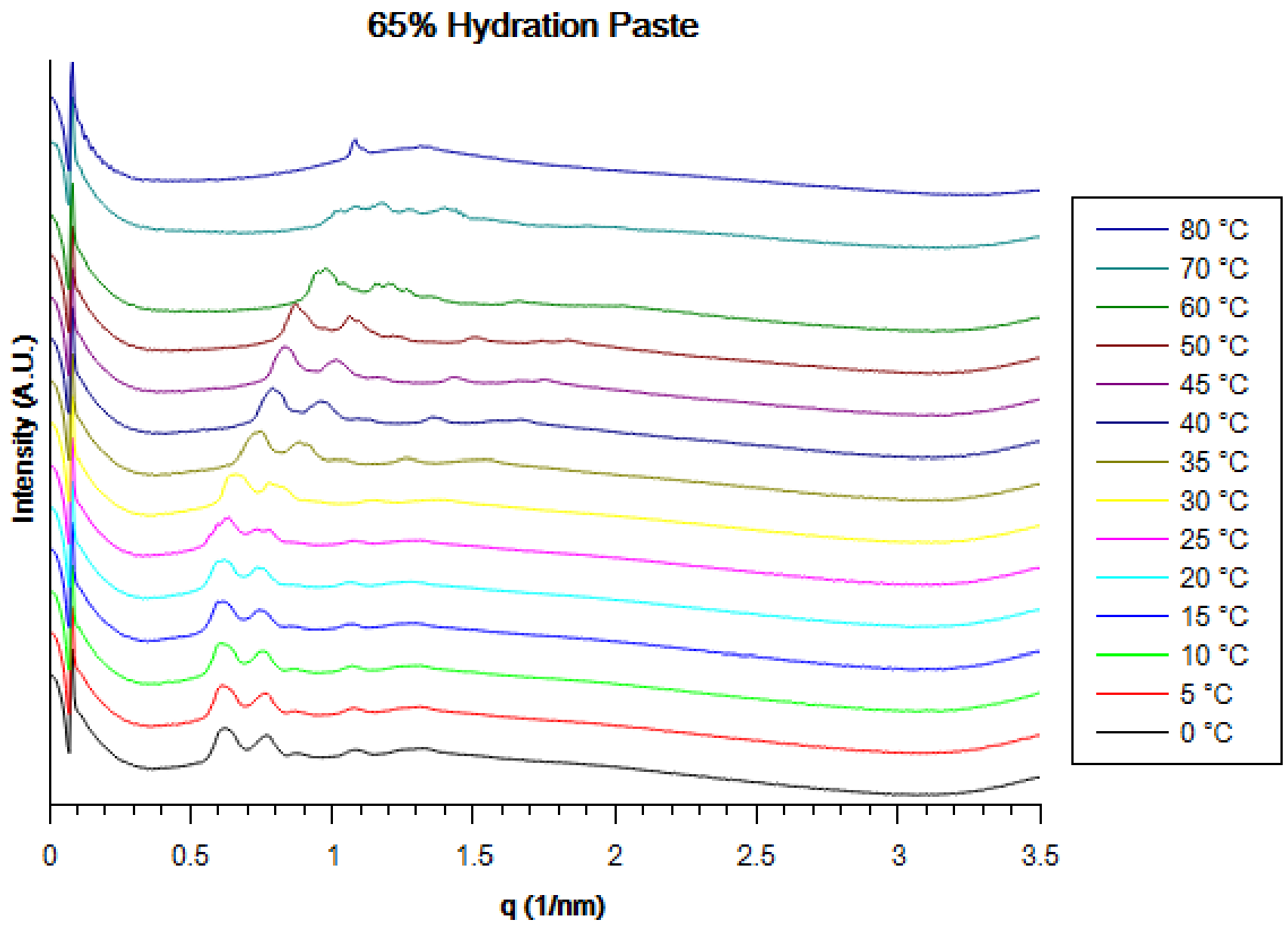

Figure S17: SAXS patterns from 65% hydration paste over the temperature range.

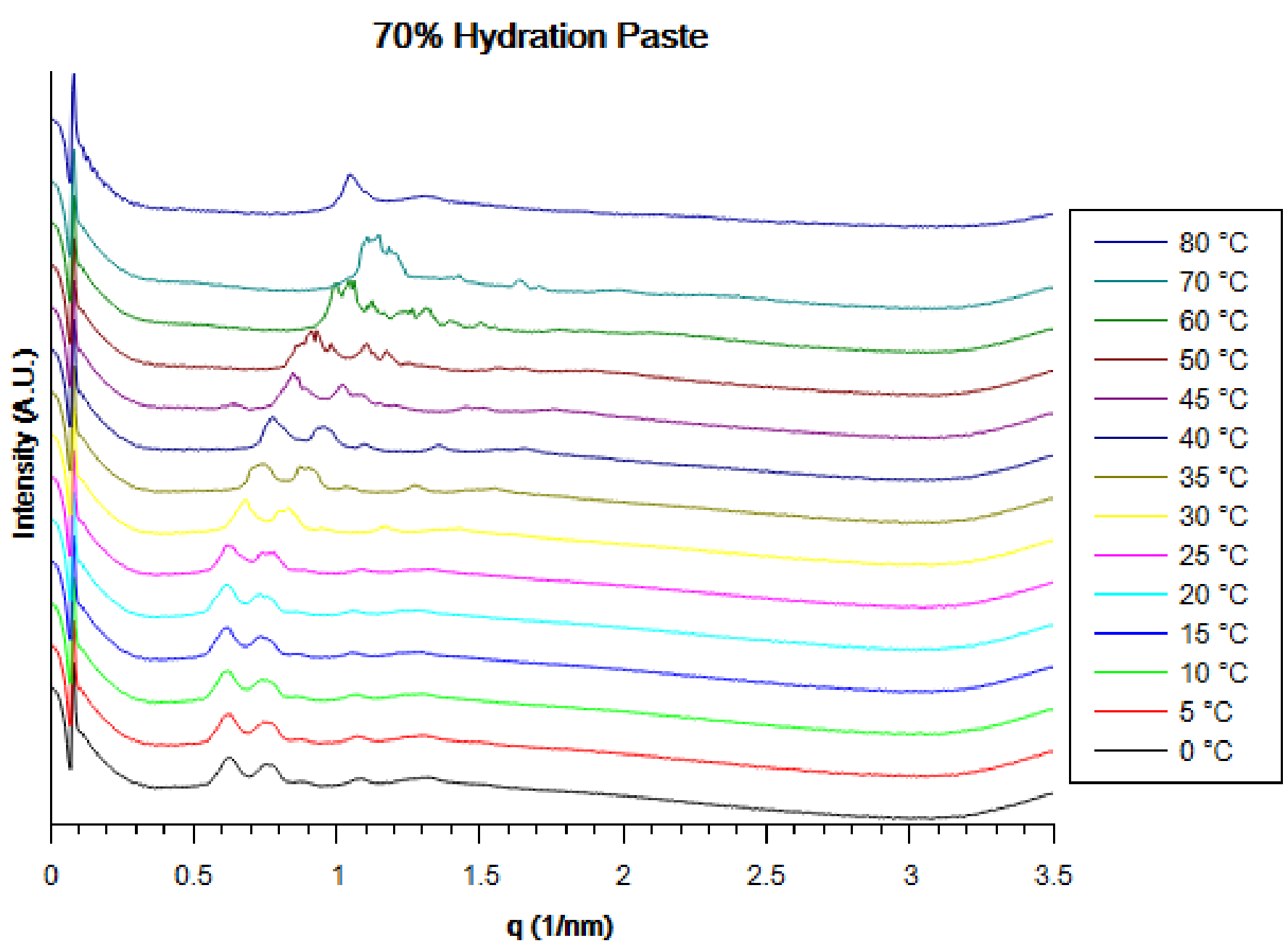

Figure S18: SAXS patterns from 70% hydration paste over the temperature range.

Below are the lattice parameter data calculated from the SAXS patterns for cubic gyroid and diamond phase.

Table S1: Lattice parameter data for gyroid and cubic phases from 0 - 25 °C.

Gyroid Lattice Parameters		Diamond Lattice Parameters	
**0 °C**			
Water content (%)	Lattice parameter (nm)	Water content (%)	Lattice parameter (nm)
25	11.65 ± 0.03	55	12.62 ± 0.06
30	11.87 ± 0.06	60	13.4 ± 0.1
35	13.63 ± 0.07	65	13.02 ± 0.06
40	14.45 ± 0.02	70	12.92 ± 0.05
45	16.04 ± 0.05		
50	18.21 ± 0.01		
**5 °C**			
25	11.43 ± 0.02	55	12.55 ± 006
30	11.53 ± 0.03	60	13.52 ± 0.03
35	13.38 ± 0.03	65	13.2 ± 0.1
40	14.24 ± 0.01	70	13.2 ± 0.1
45	15.69 ± 0.07		
50	18.22 ± 0.05		
**10 °C**			
25	11.33 ± 0.02	60	12.72 ± 0.08
30	11.33 ± 0.01	65	13.23 ± 0.03
35	13.25 ± 0.04	70	13.24 ± 0.08
40	14.04 ± 0.03		
45	15.57 ± 0.04		
50	17.78 ± 0.09		

Gyroid Lattice Parameters		Diamond Lattice Parameters	
**15 °C**			
Water content (%)	Lattice parameter (nm)	Water content (%)	Lattice parameter (nm)
25	11.11 ± 0.02	65	13.2 ± 0.1
30	11.22 ± 0.03	70	13.26 ± 0.01
35	13.11 ± 0.02		
40	13.83 ± 0.05		
45	15.49 ± 0.07		
50	17.57 ± 0.03		
**20 °C**			
25	11.01 ± 0.01	65	13.43 ± 0.02
30	11.05 ± 0.02	70	13.18 ± 0.05
35	12.94 ± 0.03		
40	13.69 ± 0.2		
45	15.2 ± 0.1		
50	17.0 ± 0.1		
**25 °C**			
25	10.88 ± 0.02	55	12.27 ± 0.05
30	10.94 ± 0.03	70	12.90 ± 0.03
35	12.77 ± 0.02		
40	13.40 ± 0.04		
45	14.78 ± 0.05		
50	16.46 ± 0.03		

Table S2: Lattice parameter data for gyroid and cubic phases from 30 - 70 °C.

Gyroid Lattice Parameters		Diamond Lattice Parameters	
**30 °C**			
Water content (%)	Lattice parameter (nm)	Water content (%)	Lattice parameter (nm)
25	10.74 ± 0.03	55	12.1 ± 0.2
30	10.72 ± 0.03	70	12.16 ± 0.08
35	12.63 ± 0.04		
40	13.14 ± 0.03		
45	14.4 ± 0.2		
50	16.0 ± 0.1		
**35 °C**			
25	10.57 ± 0.03	55	11.21 ± 0.04
30	10.58 ± 0.04	60	11.55 ± 0.04
35	12.39 ± 0.02	70	11.2 ± 0.1
40	12.88 ± 0.03		
45	13.63 ± 0.04		
50	15.15 ± 0.09		
**40 °C**			
25	10.34 ± 0.04	55	10.43 ± 0.07
30	10.24 ± 0.05	60	10.70 ± 0.02
35	12.12 ± 0.05	65	10.37 ± 0.06
40	12.514 ± 0.001	70	10.6 ±0.1
45	13.0 ± 0.1		
50	14.36 ± 0.03		

Gyroid Lattice Parameters		Diamond Lattice Parameters	
**45 °C**			
Water content (%)	Lattice parameter (nm)	Water content (%)	Lattice parameter (nm)
25	10.29 ± 0.03	55	9.79 ± 0.02
35	11.96 ± 0.01	60	10.18 ± 0.03
40	12.15 ± 0.03	65	9.84 ± 0.02
45	12.28 ± 0.09		
**50 °C**			
35	11.72 ± 0.04	50	8.03 ± 0.09
40	11.77 ± 0.07	55	9.42 ± 0.07
45	11.65 ± 0.06	60	9.64 ± 0.06
		65	9.35 ± 0.05
**60 °C**			
		35	6.91 ± 0.03
		55	8.51 ± 0.06
**70 °C**			
		35	6.94 ± 0.05
		55	7.85 ± 0.04
		60	7.97 ± 0.04
